# A non-randomized pre-post pilot study of cooling bed sheets in hot sleeping people

**DOI:** 10.3389/frsle.2025.1587801

**Published:** 2025-09-16

**Authors:** Matthew D. Weaver, Salim Qadri, Chidera Ejikeme, Stuart F. Quan, Charles A. Czeisler, Rebecca Robbins

**Affiliations:** ^1^Division of Sleep and Circadian Disorders, Department of Medicine, Mass General Brigham Hospital, Boston, MA, United States; ^2^Division of Sleep Medicine, Harvard Medical School, Boston, MA, United States

**Keywords:** sleep disruption, temperature, bedding, sleep quality, night sweats

## Abstract

**Introduction:**

Sleeping hot is a common barrier to good sleep. Characteristics of the sleep environment may impact temperature regulation and sleep. We tested the effectiveness of one brand of bed sheets that advertise cooling properties on sleep and vasomotor symptoms.

**Methods:**

Participants were recruited through multiple channels that included potential customers of the intervention sheets and targeted online advertisements. Participants completed a baseline questionnaire, daily electronic diary for 6 weeks, and an end-of-study questionnaire. Assessments included the Pittsburgh Sleep Quality Index and Restorative Sleep Questionnaire. Daily diaries assessed sleep, mood, and perceived temperature during sleep. Within-person responses were compared before and after use of the intervention bed sheets.

**Results:**

64 participants provided 2,627 total days of data. The study sample was 89% female, mean age 48 (SD 12). Sixty-nine percent of participants reported improved sleep quality after implementing the intervention. Mean improvement on the Pittsburgh Sleep Quality Index was 1.9 (95% CI 1.3–2.6), from 8.0 (SD 3.0) to 6.1 (SD 2.5) at end-of-study. The proportion of participants reporting trouble sleeping due to feeling too hot was reduced from 82.5 to 39.7%. Reported sleep duration increased 26 min (95% CI 14–38 min), from 6.5 h (SD 1.0) to 7.0 h (SD 0.8). Participants also reported improvements in night sweats, restorative sleep, mood, and alertness.

**Conclusion:**

Individuals reported improvements on several dimensions of sleep health, reductions in night sweats, and less sleep disruption due to sleeping too hot after implementing the intervention bed sheets. These findings warrant replication in a randomized, placebo-controlled design.

## Introduction

Good sleep health is a necessary prerequisite for optimal physical, cognitive, social, and mental health. While the majority of United States adults agree that getting good sleep is a major priority (Gallup, 2022a), approximately half report trouble falling asleep, staying asleep, or both (Gallup, 2022b); and 40% describe the quality of their sleep as poor (Quan et al., 2023). One of the most common reasons individuals report poor sleep is feeling too hot (Gallup, 2023).

During sleep, we normally radiate heat from our skin, particularly the hands, feet, and forehead, in order to lower core body temperature. This may be accompanied by sweating, particularly if we are under heavy blankets or if the ambient temperature is warm. Even though core body temperature is dropping, because skin temperature is rising, many individuals consider themselves “hot sleepers.” Moreover the ability to thermoregulate is attenuated during sleep, in particular during REM sleep (Heller et al., 1988). Therefore, ambient temperature and the temperature of the sleep environment can present significant barriers to healthy sleep (Obradovich et al., 2017; Deng et al., 2024). In addition, night sweats and other vasomotor symptoms are a common experience for many adults due to a variety of factors, including medications, inflammation, the menopausal transition, and chronic disease, such as obstructive sleep apnea and blood dyscrasias (Mold et al., 2012).

One strategy to mitigate the impact of thermal environments on sleep health is curating a healthy sleep microenvironment. For example, mattress composition, firmness, and cooling may impact sleep (Radwan et al., 2015; Li et al., 2021; Robbins et al., 2025); as do the textiles and fabrics used in bed sheets and bedding materials (Li et al., 2024; Pasquier et al., 2024; Shin et al., 2016). Marketing materials for sheets and comforters often extol the benefits of cooling elements in these products. However, research on the effectiveness of these products is limited, particularly among adults experiencing vasomotor symptoms such as night sweats. We sought to test the impact of a specific brand of bed sheets that advertise cooling properties on multiple dimensions of sleep and daytime function, with a particular emphasis on the frequency of sleep disruption attributed to sleeping too hot.

## Methods

We conducted a within-person before and after assessment of cooling bed sheets manufactured by Lusomé (Calgary, AB, Canada). Recruitment was conducted through multiple channels. The recruitment strategy targeted: 1) individuals who had at one time added the product to their online shopping cart and provided their contact details, but did not actually purchase the bed sheets; 2) prior purchasers of other products from the company; 3) emails to members of The Sweaty Pillow Community (an online community of individuals navigating menopause); and 4) targeted digital advertisements designed to reach women 40–55 years of age who were interested in topics related to health and menopause. Those who expressed interest in participating completed a brief screening questionnaire. Eligibility criteria required participants to report feeling too hot while sleeping at night and having access to air conditioning systems in their sleeping area. Participants were ineligible if they had previously purchased the study product, were currently using a cooling mattress topper, or had a history of one or more sleep disorders. Individuals who met inclusion criteria were then offered the opportunity to participate in the study. Those who elected to do so were required to purchase the sheets, with the commitment that their purchase price (USD $250 value) would be refunded upon their completion of the study. All participants provided informed consent and accepted a delay in shipment of a new pair of bed sheets to facilitate within-person data collection (before and after) using the bed sheets. The intervention sheets were 100% cotton sheets treated with Xirotex™ Cool. This treatment is intended to absorb and store heat before sweating occurs.

Following consent, participants were sent a baseline survey that included a series of validated questionnaires. Sleep quality and sleep duration were assessed using the Pittsburgh Sleep Quality Index (Buysse et al., 1989). Restorative sleep was assessed using the REST-Q (Robbins et al., 2022). Two items from the Patient-Reported Outcomes Measurement MenoScores Questionnaire were also included to characterize vasomotor symptoms (e.g., hot flashes and/or night sweats; Lund et al., 2018). Respondents were invited to complete an end-of-study assessment 4 weeks after receiving their bed sheets. The end-of-study assessment contained the same instruments as the baseline survey, facilitating pre-post comparisons.

All participants also completed a daily electronic sleep diary once per day throughout the study interval. A field on the electronic diary enabled users to indicate when the intervention bed sheet set had arrived and when the participant began to use the intervention. The daily diary included electronic momentary assessments for temporally dynamic outcomes, including current mood, energy levels, and perceived stress. The total study duration was 6 weeks (2 weeks prior to the intervention and 4 weeks after).

Primary comparisons for sleep duration, sleep quality, and sleep efficiency were conducted through comparison of the baseline and end-of-study survey. Mood and wellbeing assessments were collected using the daily electronic diary. Sleep disruption due to sleeping too hot was compared using both the baseline and end-of-study assessments as well as the daily electronic diary.

We tested the change in PSQI global score as a continuous variable. The mean difference before and after the intervention and corresponding 95% confidence interval was calculated. We also estimated the proportion of participants reporting improvement in PSQI, the proportion with a clinically meaningful reduction (≥3; Buysse et al., 2011), and the proportion transitioning from a score corresponding to poor sleep quality (≥5) to a score below that threshold (Buysse et al., 1989). The specific PSQI item, “During the past month, how often have you had trouble sleeping because you feel too hot” was also examined in depth. Response options include “Not during the past month,” “Less than once a week,” “Once or twice a week,” and “Three or more times a week.” We examined the change in the proportion of respondents reporting trouble sleeping due to sleeping too hot at least once a week by combining responses of “Once or twice a week” or “Three or more times a week.”

Response options for the MenoScores questionnaire item, “I have not been able to sleep because of night sweats” included “No, not at all,” “Yes, a bit,” “Yes, quite a bit,” and “Yes, a lot.” We examined changes in frequency of night sweats among those who reported any of the “Yes” options at baseline. Recording the same response at end-of-study was considered neutral. The proportion with a reduction in frequency was reported (e.g., moving from a response of “Yes, quite a bit” to “Yes, a bit”). We also reported the proportion who responded “No, not at all” at the end-of-study (symptom eliminated).

Mood and wellbeing were characterized on the daily diaries using visual analog scales (response options 0–100). The scales were separately anchored by axis labels of Sleepy-Alert, Sad-Happy, Sluggish-Energetic, Sick-Healthy, Stressed Out-Calm Relaxed, and Sleep Hot-Sleep Cold. Subject mean responses were estimated for the days participants' contributed data before and after initiating the intervention. We tested the difference in mean values pre- and post-intervention.

Continuous variables that met parametric assumptions were compared using paired *t*-tests. Non-parametric comparisons were conducted using the Wilcoxon signed-rank test. The McNemar test was used for tests of paired proportions. Likelihood of Type I error was set at 0.05. Two-sided tests were using for all comparisons. The statistical analysis was carried out in Stata V15 SE (College Station, TX).

## Results

Two hundred fifty-five individuals expressed interest in the study, 143 of whom were determined to be eligible. In total, 64 participants consented to participate and completed the study assessments before and after receiving the intervention bed sheet set (Table 1), contributing 2,627 total days of data to the analysis.

**Table 1:** Demographic characteristics of the study participants.

**Characteristic**	**Proportion *(n)* 100% (64)**
**Age, mean (SD)**	48 (SD 12)
**Female sex**	89% (57)
**Body mass index**	
Underweight (<18.5)	2% (1)
Normal weight (≥18.5 and <25)	53% (34)
Overweight (≥25 and <30)	30% (19)
Obese (≥30)	16% (10)
**Race**	
White	83% (53)
Asian	13% (8)
Black or African American	2% (1)
Other	3% (2)
**Household income**	
<$100,000	36% (23)
$100,000–$149,999	14% (9)
$150,000–200,000	27% (17)
≥$200,000	23% (15)
**Highest level of education**	
Less than undergraduate degree	16% (10)
Undergraduate degree	38% (24)
Graduate Degree	46% (29)

^*^One participant is missing household income and education (*n* = 63).

### Sleep quality

The mean PSQI score at baseline was 8.0 (SD 3), with 75% reporting poor sleep quality (PSQI score >5; Table 2). Sixty-nine percent of participants reported a reduction (improvement) in PSQI scores after implementing the intervention. The mean change in PSQI score between the baseline and end-of-study assessment was a reduction of 1.94 (95% CI 1.31–2.56; *p* < 0.001). As shown in Table 3, analyses of changes in individual components of the PSQI found that those representing Duration, Sleep Disturbance, Sleep Latency and Sleep Efficiency improved at the end-of-study assessment. There was no difference in daytime dysfunction, the single-item measure of overall sleep quality, or the use of sleep medications. Thirty-eight percent of participants reported a PSQI reduction ≥3, which is the threshold considered to be clinically meaningful.

**Table 2:** Baseline vs. end-of-study comparisons from the PSQI, PROMS, and restorative sleep questionnaire.

**Baseline and end-of-study assessments**	**Baseline**	**End-of-study**	***P*-value**
PSQI score (mean, SD)	8.0 (3.0)	6.1 (2.5)	<0.001
Poor sleep quality [PSQI score >5 *(%, n)*]	75% (48)	48% (31)	0.03
Time in bed (mean, SD)	8.1 h (1.1)	8.4 h (0.9)	0.02
Sleep latency (mean, SD)	28.1 min (20.0)	14 min (11.9)	<0.001
Sleep duration (mean, SD)	6.5 h (1.0)	7.0 h (0.8)	<0.001
Sleep efficiency (mean, SD)	81.6% (11.3)	84.1% (10.3)	0.08
Trouble sleeping due to feeling too hot (%, *n*)^*^	82.5% (52)	39.6% (25)	<0.001
REST-Q score (mean, SD)	24.8 (7.6)	31.1 (7.1)	<0.001

^*^One participant did not report the frequency of sleeping too hot at baseline. Estimates include the 63 participants who reported the frequency at both time points.

**Table 3:** PSQI component scores at baseline, end-of-study, and the observed difference.

**PSQI component**	**Baseline component score Mean (95% CI)**	**End-of-study component score Mean (95% CI)**	**Mean difference (95% CI)**	***P*-value**
Duration	1.39 (1.22-1.56)	0.97 (0.84-1.10)	**–**0.42 (−0.60 to −0.24)	<0.001
Sleep disturbance	1.42 (1.20–1.64)	0.77 (0.58–0.95)	−0.66 (−0.86 to −0.45)	<0.001
Sleep latency	1.03 (0.88–1.19)	0.81 (0.69–0.94)	−0.22 (−0.36 to −0.07)	0.001
Daytime dysfunction	0.89 (0.65–1.13)	0.70 (0.47–0.93)	−0.19 (−0.44 to −0.07)	0.15
Sleep efficiency	1.70 (0.58–1.83)	1.38 (1.25–1.50)	−0.33 (−0.49 to −0.17)	<0.001
Overall sleep quality	0.44 (0.20–0.67)	0.37 (0.15–0.59)	−0.06 (−0.19 to 0.06)	0.29
Sleep medications	1.16 (1.05–1.27)	1.11 (1.03–1.19)	−0.05 (−0.16 to 0.07)	0.41
**Total PSQI score**	8.02 (7.26–8.77)	6.08 (5.46–6.70)	−1.94 (−1.31 to −2.56)	<0.001

Seventy-five percent of participants (*n* = 48) reported poor sleep quality at baseline (Table 2). Among these participants, the mean change in PSQI score was a reduction of 3.8 (95% CI 2.9–4.6; *p* < 0.001). Of those reporting poor sleep quality at baseline, 44% no longer reported poor sleep quality at the follow-up assessment (*p* = 0.03).

### Sleeping too hot

At baseline, most participants reported trouble sleeping due to feeling too hot at least once a week (82.5%). The fraction of participants reporting sleeping too hot at least once a week was (39.7%) at the follow-up assessment (*p* < 0.001; Table 2).

Information regarding sleeping too hot was also collected on the daily electronic diary. Participants completed 14 daily diaries on average (SD 1.7) prior to the intervention and 27 days (SD 1.8) after implementing the intervention. Participants rated their sleep from the previous night on a continuum from sleeping hot (0) to sleeping cold (100). The mean rating prior to the intervention was 32.9 (95%CI 26.7–37.1). The mean rating following the intervention was 52.2 (SD 95%CI 47.6–56.8); corresponding to a mean within-subject difference of 19.3 (95%CI 14.8–23.9; *p* < 0.001) (Table 4).

**Table 4:** Daily diaries before intervention vs. daily diaries during intervention interval.

**Daily diary assessments**	**Pre-intervention Mean (95% CI)**	**During-intervention Mean (95% CI)**	***P*-value**
Sleeping cold (100) vs. sleeping hot (0)	32.9 (28.7–37.1)	52.2 (47.6–56.8)	<0.001
Alert (100) vs. sleepy (0)	47.3 (41.5–53.0)	55.4 (49.8–61.0)	<0.001
Energetic (100) vs. sluggish (0)	45.1 (39.6–50.6)	55.8 (50.3–61.3)	<0.001
Happy (100) vs. sad (0)	66.4 (61.0–71.7)	70.8 (66.5–75.1)	0.02
Healthy (100) vs. sick (0)	71.9 (66.4–77.3)	73.0 (68.2–77.8)	0.49
Calm-relaxed (100) vs. stressed out (0)	60.9 (55.2–66.6)	63.6 (58.7–68.5)	0.15

**Figure 1:**
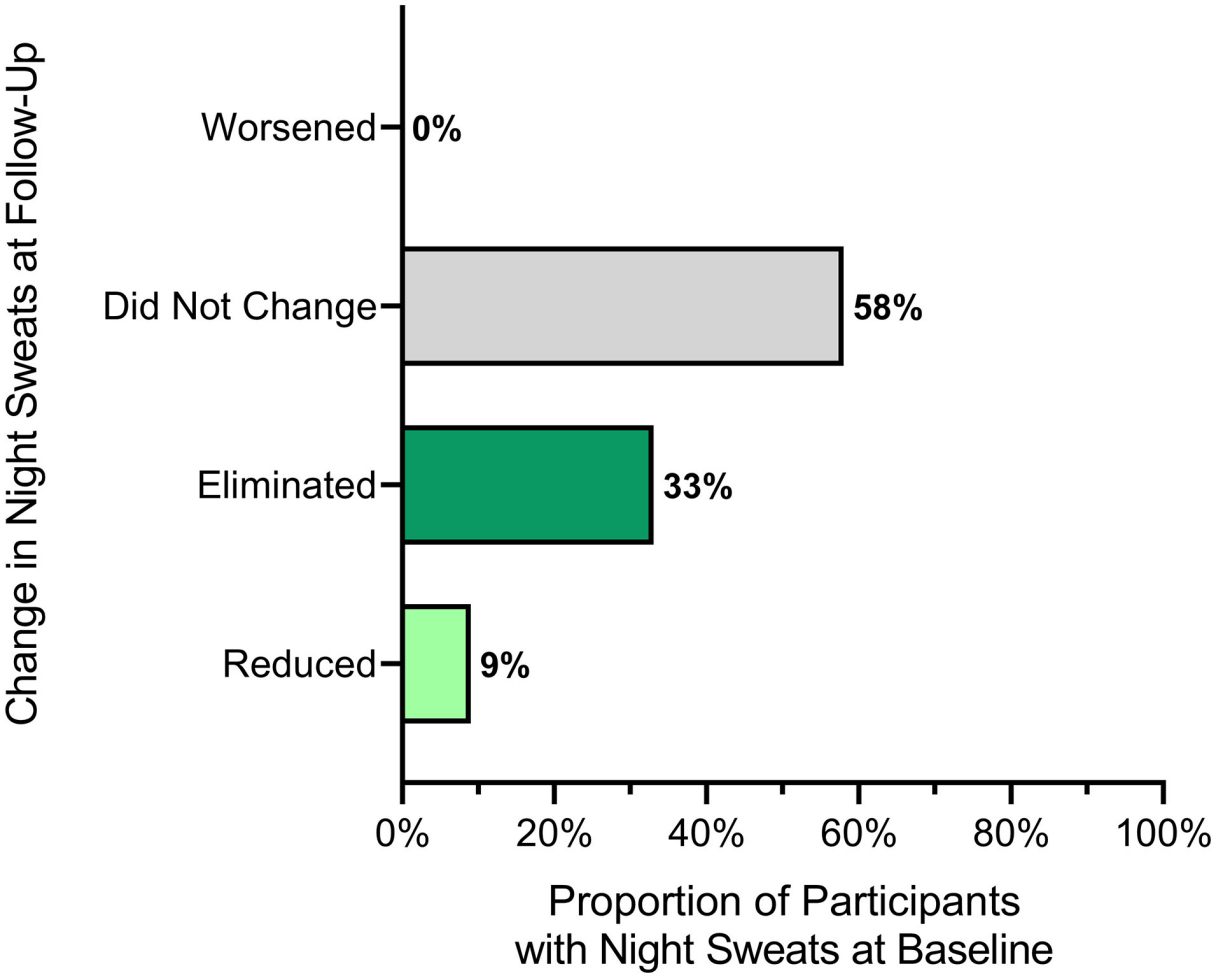
The change in the proportion of participants reporting inability to sleep due to night sweats at the end-of-study compared to the baseline questionnaire. This figure illustrates changes in responses to the question, “I have not been able to sleep because of night sweats” between the end-of-study and the baseline questionnaire. Individuals reporting the same level of disruption were characterized as neutral. Individuals who reported a reduction in frequency are included in reduced. Individuals who reported any level of disruption at baseline and denied any problem at the end-of-study are included in the eliminated category.

The prevalence of difficulty sleeping due to night sweats was 75% (*n* = 48) at the baseline assessment. Among those reporting these symptoms at baseline, 42% reported a reduction in the severity of night sweat symptoms at the follow-up assessment (*n* = 20/48), with the symptom eliminated for 33% (*n* = 16/48; *p* < 0.001; Figure 1).

### Time in bed, sleep latency and sleep duration

Participants reported an average of 8.1 h (SD 1.1) time-in-bed at baseline that included 6.5 h (SD 1.0) sleep duration. Participants reported more time-in-bed after using the cooling sheets (mean difference 19 min, 95% CI 3–36 min, *p* = 0.02), reduced sleep latency (mean difference 14 min, 95% CI 10–18 min, *p* < 0.001), increased sleep duration (mean difference 26 min, 95% CI 14–38 min, *p* < 0.001; Table 2).

### Sleep efficiency

Sleep efficiency averaged 81.6% (SD 11.3%) at baseline and 84.1% (SD 10.3%) at follow-up, for a mean difference of 2.5% (95% CI −0.3–5.3%). This comparison did not reach statistical significance (*p* = 0.08; Table 2), though the PSQI component score for sleep efficiency was improved (Table 3).

### Restorative sleep

The baseline mean score for the REST-Q was 24.8 (SD 7.6). The mean REST-Q score at follow-up was 31.1 (SD 7.0), corresponding to a mean increase of 6.3 (95% CI 4.3–8.3; *p* < 0.001; Table 2).

### Mood and wellbeing

Electronic momentary assessments on the daily diary revealed that daily mean assessments of alertness significantly increased after the intervention (*p* < 0.001) (Table 3), participants reported feeling more energetic (*p* < 0.001), and reports of mean happiness were higher (*p* = 0.02). There was no change on the assessment of Sick-Healthy (*p* = 0.49) or mean daily stress levels (*p* = 0.15; Table 4).

## Discussion

Healthy sleep offers myriad benefits across many domains of health. One of the well-documented barriers to sleep are issues with temperature regulation, namely, individuals who identify as hot sleepers. While sleep disruption due to feeling too hot or experiencing night sweats occurs among all sexes and age groups for a variety of reasons (Mold et al., 2012; Lack et al., 2008; Larnard et al., 2023), vasomotor symptoms are of particular concern among women during the menopausal transition (Kravitz et al., 2008); and have an additive effect on the burden of adverse mental health outcomes (Joffe et al., 2016). Although store shelves are full of products that claim to attenuate temperature issues and promote better sleep, few have been tested. We conducted a within-person pilot study of a specific brand of bed sheets that advertise cooling properties among men and women who identify as hot sleepers.

Results of this pilot study reveal that, after introducing the cooling bed sheets, there was a significant improvement in sleep quality. In addition, the proportion of individuals reporting sleeping hot at least once per week was cut in half after introducing the bed sheets, as did sleep difficulty complaints that were reported to be due to night sweats. After introducing the bed sheets, participants reported being able to sleep on average ~25 min longer than before introducing the bed sheets. We also observed improvements in restorative sleep scores. Finally, responses to visual analog scales indicated participants felt more energetic, happier, and less stressed during the day after introducing the sheets. Our findings suggest that a non-pharmacological intervention can improve the sleep of persons who feel that their sleep is impaired because they feel too “hot” or who suffer from vasomotor symptoms at night. Such persons are often prescribed various medications which may have attendant adverse effects. Cooling bed sheets may be a worthwhile alternative.

Our study was not designed to inform the mechanisms by which the intervention provided benefit. However, it is possible that the bedsheets facilitated heat loss resulting in a further reduction in the normal decline in core body temperature (CBT) during nocturnal sleep. Lowering of CBT is associated with an increase in sleep wave (N3) sleep which in turn has been linked to better subjective sleep quality (Togo et al., 2007). In support of this hypothesis, use of a high-heat capacity mattress has been demonstrated to increase N3 sleep (Herberger et al., 2024). In contrast, a recent systematic review and meta-analysis of nine studies found no impact of types of cooling bedding (i.e., mattress, mattress topper) on sleep architecture, although there were no studies using bedsheets (Pasquier et al., 2025). Alternatively, participants may have experienced a subjective improvement in their sleep environment related to the bedsheets inducing a soothing contact with the skin or a sensation of coolness. Our findings of improvement in subjective restorative sleep, mood and wellbeing suggests this may have occurred.

There are several important limitations to this study. First, we employed a non-randomized, pre-post design where participants were not blinded to the intervention. Thus, the lack of a control group and the impact of temporal changes could have explained some of our findings. Second, we recruited participants who already had indicated interest in purchasing the cooling bed sheets and may have had an a priori favorable opinion of them. Given that participants were recruited from brand-affiliated communities and initially purchased the bed sheets, there is a potential for bias in that they may have internalized some loyalty to the company or its mission, and have pre-existing expectations of a positive impact from using the product (Hirschman, 1970). However, we sought to mitigate this by the provision of a full refund for the purchase price of the product once they completed the study. Third, we studied one manufacturer's cooling bed sheet product. Whether our results are generalizable to other similar bedding products or other manufacturer's products remains to be determined. Fourth, our eligibility criteria required that individuals interested in participating reported feeling too hot while sleep at night. Our study was conducted in Summer and Fall months in the Boston Area (June–October 2024). Therefore, we cannot determine whether there would be seasonal variation in our results. Fifth, 90% of our participants self-identified as female. They also were primarily White race, middle aged, college educated and had an above average income. Therefore, they are not representative of the general U.S. adult population. Lastly, we relied on subjective reports of sleep, which often differ from objective assessments (Baker et al., 1999; Lockley et al., 1999; Lauderdale et al., 2008), and we did not collect information on whether or not participants changed other components of the sleep environment (e.g., pillows or blankets) during the study interval. Nevertheless, despite the above limitations and the relatively small sample size of the study population, our results demonstrating better sleep and improvement in “hot” sleeping are striking and suggest a beneficial impact of cooling sheets.

In summary, those suffering from hot sleeping complaints appear to benefit from the introduction of cooling bed sheets. Our study revealed improvements in several sleep parameters as well as daytime performance assessments. Future research is needed to confirm these results in a placebo controlled, randomized study design.

## Data Availability

The datasets presented in this article are not readily available because our ethical approval prohibits data sharing without a mutually executed Data Use Agreement or acceptable equivalent approved by the Mass General Brigham Contracting Office. Requests to access the datasets should be directed to mdweaver@bwh.harvard.edu.
